# Welcoming a new Main Editor of *Journal of Synchrotron Radiation*

**DOI:** 10.1107/S1600577524005733

**Published:** 2024-06-25

**Authors:** Andrew J. Allen, Dibyendu Bhattacharyya, Kristina Kvashnina

**Affiliations:** a National Institute of Standards and Technology 100 Bureau Drive Gaithersburg MA20899 USA; b Atomic and Molecular Physics Division Bhabha Atomic Research Centre Mumbai400085 India; c Rossendorf Beamline, ESRF – The European Synchrotron, 71 Avenue des Martyrs, 38000Grenoble, France

**Keywords:** *Journal of Synchrotron Radiation*

## Abstract

Introducing a new Main Editor of *JSR*.

Makina Yabashi of RIKEN SPring-8 Center, Japan, has recently been appointed as a Main Editor of *Journal of Synchrotron Radiation*. Dr Yabashi received his bachelor’s and master’s degrees from the University of Tokyo, Japan. After a short period as a PhD student, he joined the SPring-8 construction project, focusing on X-ray beamline development, as a researcher of JASRI in 1996. He particularly contributed to the construction of the 25 m undulator beamline 19LXU, where he developed an ultrahigh-resolution X-ray monochromator using asymmetric reflections and conducted Hanbury Brown and Twiss experiments in the hard X-ray region, enabling diagnostics of a small vertical emittance of the SPring-8 storage ring. He received his PhD degree from the University of Tokyo in 2003, and then joined the project to develop the SCSS test accelerator, a prototype machine of the compact X-ray free-electron laser (XFEL) source at SPring-8, later named SACLA. He then completed a diagnostic system on EUV radiation, contributing to the early success of the lasing of SCSS. In the SACLA construction project in 2006–2011, Dr Yabashi led the development of XFEL beamlines with dedicated X-ray optics, diagnostics and experimental methods and instruments. In 2011, he was appointed Director of the Beamline Research and Development Group of RIKEN SPring-8 Center (RSC), where he has taken the initiative in R&D to exploit the unique capabilities of SACLA and promote scientific applications. In 2018, he held the concurrent position of Director of the Physical and Chemical Research Infrastructure Group of RSC, leading the refurbishment and upgrade of SPring-8 beamlines. In 2024, he was appointed Deputy Director of the Strategic Office for the SPring-8-II Project of RSC. Dr Yabashi has received many awards including the MEXT Commendation for Science and Technology, Japan, in 2019. Since 2016, he has been co-organizing the XOPT international conference series in Yokohama as Chair of the Program Committee, and has also served as a member of many international review committees, such as Swiss­FEL FLAC, LCLS SAC, PAL IAC and PSI PSAC. ▸

Dr Yabashi has been active as a Co-editor of *Journal of Synchrotron Radiation* since 2014. We are delighted to welcome Dr Yabashi to the team of Main Editors, and look forward to working with him to further develop the journal for the synchrotron radiation and free-electron laser communities. We would also like to thank Professor Yoshiyuki Amemiya for his many years of service as a Co-editor and Main Editor of *Journal of Synchrotron Radiation*, and wish him well as he steps down at this time.

## Figures and Tables

**Figure 1:**
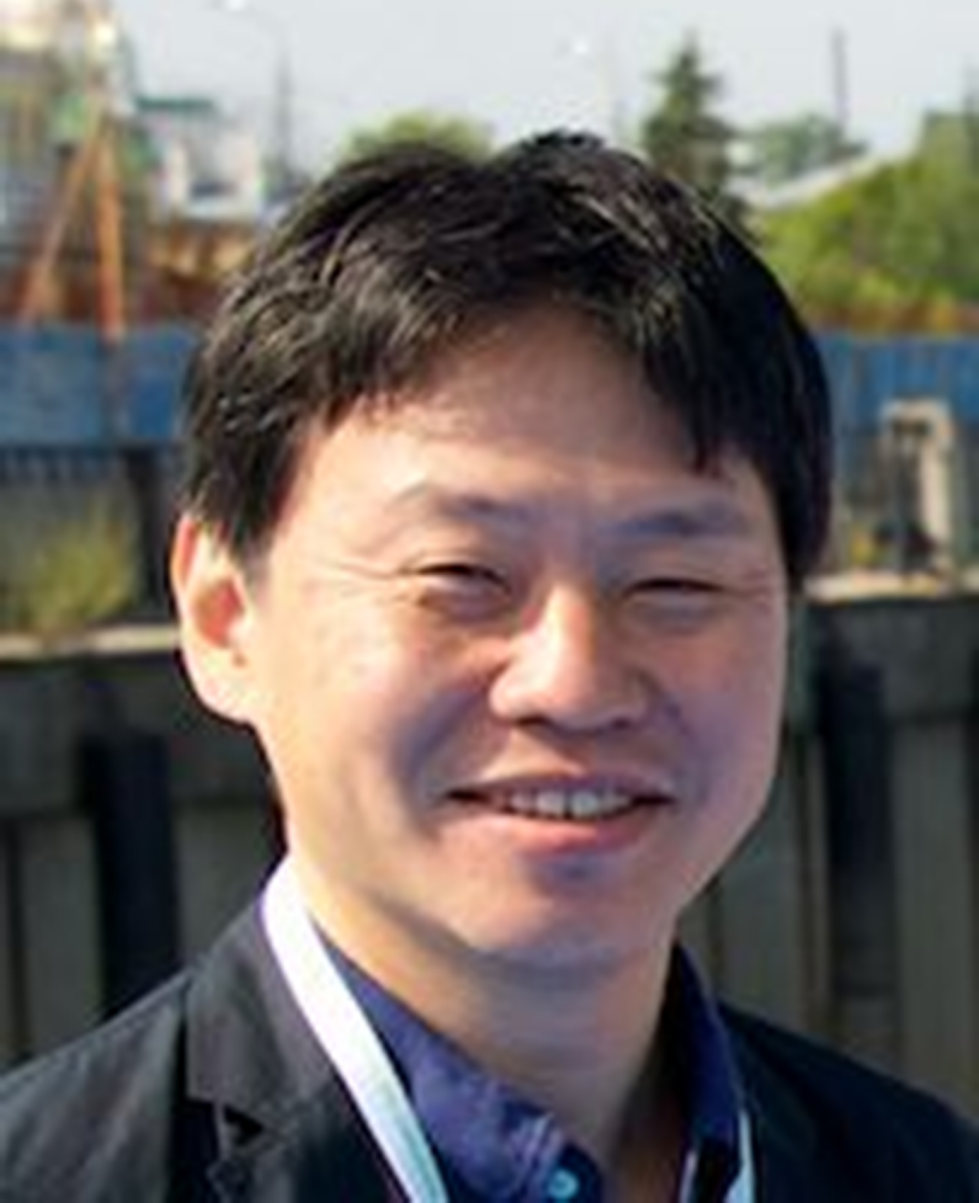
Dr Makina Yabashi, new Main Editor of *Journal of Synchrotron Radiation*.

